# Conservation tillage (CT) for climate-smart sustainable intensification: Benchmarking CT to improve soil properties, water footprint and bulb yield productivity in onion cultivation

**DOI:** 10.1016/j.heliyon.2024.e39749

**Published:** 2024-10-23

**Authors:** Md Mashiur Rahman, Naznin Sultana, Muhammad Arshadul Hoque, Md. Golam Azam, Md. Rafiqul Islam, Md. Altaf Hossain

**Affiliations:** a Agricultural Engineering Division, Pulses Research Centre & Regional Agricultural Research Station, Bangladesh Agricultural Research Institute, Ishurdi, 6620, Pabna, Bangladesh; b Department of Agricultural and Biological Engineering, The Pennsylvania State University, University Park, 16802, PA, USA; c Entomology Division, Bangladesh Sugarcrop Research Institute, Ishurdi, 6620, Pabna, Bangladesh; d Farm Machinery and Postharvest Process Engineering Division, Bangladesh Agricultural Research Institute, Gazipur, 1701, Bangladesh; e Plant Breeding Division, Pulses Research Centre, Bangladesh Agricultural Research Institute, Ishurdi, 6620, Pabna, Bangladesh; f Agronomy Division, Regional Agricultural Research Station, Bangladesh Agricultural Research Institute, Ishurdi, 6620, Pabna, Bangladesh; g Entomology Division, Pulses Research Centre, Bangladesh Agricultural Research Institute, Ishurdi, 6620, Pabna, Bangladesh

**Keywords:** Conservation agriculture, Climate-smart agriculture, Agricultural sustainability, Soil health improvement, Water-use efficiency, Yield optimization

## Abstract

Environmental sustainability indicators for conservation tillage (CT) in agricultural systems primarily focus on assessing the impacts on soil organic carbon (SOC) and water footprint (WF). One way to improve these indicators is by boosting crop production while minimizing environmental impact through the implementation of sustainable intensification (SI) and climate-smart agriculture (CSA) to ensure food security. Conservation agriculture (CA) based CT practice with crop residue retention has potential for benchmarking the conserving of water in agriculture, improving soil health, crop productivity and ensuring agricultural sustainability. The CT-based water-saving potential in onion cultivation nonetheless remains understudied in Bangladesh. For this, a field experiment was undertaken to assess soil properties and water footprint in onion cultivation in the Charland agroecosystem in Jamalpur, Bangladesh. Three different tillage practices, such as minimum tillage (MT), tractor tillage (TT) and conventional power tillage (PT), and flatbed flood irrigation were introduced with four replications. Tillage practices showed significant positive effects on yield characteristics and bulb yields. The findings indicate that the MT practice in onion (BARI Piaz-4) cultivation resulted in the highest fresh bulb yield of 22.79 t ha^−1^, followed by TT (20.48 t ha^−1^) and PT (16.25 t ha^−1^) practices. The MT practice achieved the highest water footprint (WF) savings of 40% water, where crop biomass, including above and below-ground parts, bulb size, yield characteristics, and yield productivity were significantly increased. The findings also indicate a direct correlation between the water productivity index (WPI), WF and the bulb yield under MT practice. The study's findings favor CT practice and, therefore, suggest a methodology of employing MT practice as a benchmark to increase agricultural water-saving potential. It can also be used as a reference for promoting water conservation practices achieving sustainable development and improving resource efficiency in agriculture.

## Highlights

• Conservation tillage (CT) and water footprint (WF) are introduced in onion cultivation.
• Minimum tillage shows the highest onion bulb productivity in Charland agroecosystem.
• Water productivity index (WPI) performed highest in minimum tillage practice.
• Water footprint confirms 40 % water savings by minimum tillage practice.
• WF and CT propose a methodology to evaluate agricultural water-saving potentials.

## Graphical abstract

**Figure:**
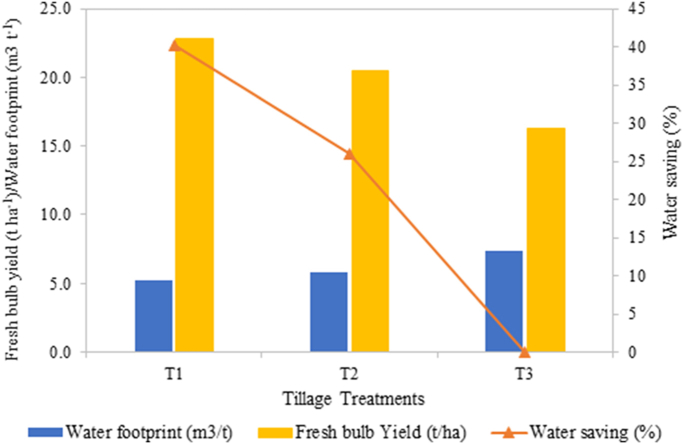

## 1 Introduction

A significant proportion of agricultural soils have become vulnerable to the rapid degradation of soil health resulting from excessive tilling [1,2]. Moreover, Soil tillage techniques have a direct impact on various soil-related variables, including soil erosion, the rate at which crop residue decomposes, soil temperature, soil structure, microbiological activity, nitrogen mineralization, soil organic carbon and the level of soil moisture reduction [3]. Nonetheless, the humid subtropical environment leads to a quick decomposition of soil organic matter (SOM) by heterotrophic microorganisms. The presence of a low SOM content is a significant factor contributing to reduced productivity in agriculture in Bangladesh, and this condition is widely recognized as a threat to the long-term viability of agricultural crop production [2,4].

In this regard, conservation agriculture (CA), specifically conservation tillage systems (CTS) with crop residue retention, is frequently recommended as a way to increase crop productivity while also preserving soil health, increasing water-saving potential and gaining agricultural sustainability [2]. The two approaches, sustainable intensification (SI) and climate-smart agriculture (CSA) technologies, are closely related and are increasingly being promoted in agricultural policy circles in Bangladesh [5]. SI is a guiding measure required for making agricultural activities more productive and sustainable, while CSA is considered as an essential mechanism for gaining the Sustainable Development Goals (SDGs) [6,7]. Moreover, the SI approach focuses on increasing food production while minimizing environmental impact, while the CSA is more concerned with building adaptive capacity and reducing potential agricultural emissions. The research findings suggest that more research is needed to explore the transformative potential of CSA beyond just technical solutions, as there is a paucity of research on how CSA can transform agriculture productivity to safeguard food security [8,9].

Onion is one of the important spice crops and is widely used as a spice mainly for preparing cooking curries in Indian subcontinent countries like Bangladesh and worldwide. Bangladesh mostly cultivates onions; however, its output rate remains far below the global average. On a global basis, the average standard of onion production is 18–20 tons per hectare.; however, the average yield in Bangladesh is 11 tons [10]. Rajshahi and Dhaka divisions have a greater productivity area for onion production. However, the recent onion crisis in Bangladesh has led to a sustainable plan and management scope to adopt new technology to increase onion cultivation [11]. One approach to enhance onion productivity is by implementing effective strategies on the fellow lands, adapting the most productive variety and climate-smart conservation technologies [12]. Therefore, there is still hope to increase onion production in the fallow Charland agroecosystem in Bangladesh, especially the Jamalpur region, by adopting sustainable technologies, such as CA technologies, CSA technologies, and tilling machinery.

Conservation tillage (CT) practice is one of the CA and CSA technologies that act, while cultivating the soil to enhance its air, enabling the infiltration of moisture and aeration - this facilitates seed germination, promotes root development, manages weed expansion, and facilitates incorporation of fertilizers into the soil resulting in improved soil and crop yield productivity [2,13]. Researchers reported that crop yield can be increased up to 20–30 % by adopting suitable irrigation practices along with CT practices [14]. CT options could be essential for higher productivity and profitability in onion cultivation [15,16]. Soil preparation and soil type have greatly affected seedling transplanting to vegetative production and, finally, the formation of the onion bulb.

Conversely, watering the soil is essential for many reasons, including retaining moisture and air, promoting root development, germination of seeds, and solubilizing fertilizers to available forms in the soil. In flatbed onion production in Bangladesh, the main method of watering is furrow (flood) irrigation [17,18]. By flooding the beds in the furrows, the plants are able to absorb water gradually and thoroughly using the furrow irrigation method. Onions have a shallow root system, with approximately 90% of their roots concentrated in the uppermost 20 cm of the soil. Since onions have a shallow root system, early bulb development may be encouraged by insufficient watering, resulting in smaller onions and reduced crop yield. The frequency and availability of irrigation will vary based on factors such as soil type, crop growth stage, weather conditions and irrigation system type. It is advisable to employ regular and gentle irrigation prior to establishment of the crop [19]. The crucial time for irrigation begins at the plant's establishment and continues until the bulb expansion stage. Soils with lower density require more frequent watering but with a lower volume of water per application [19]. Studies have demonstrated that onion roots only develop at the stem plate when moisture is present, and effective moisture control is crucial for maintaining optimal root development, promoting bulb growth, and enhancing overall vitality. Watering should be stopped once the bulbs have achieved their maximum size and the tops have started to turn brown (at least two weeks before lifting) [20].

At present, there is no technology accessible that can employ synchronized conservation tillage and irrigation methods for benchmarking agricultural water-saving potentials in the cultivation of onion during the Rabi season. Therefore, there may be potential to enhance onion production in the Charland agroecosystem by using sustainable agricultural practices, including CT practice, sustainable irrigation practice, residue management and mulching. Therefore, this research aimed to determine the impact of different tillage practices, water-saving potential, and water footprint in onion cultivation on bulb yield characteristics and productivity. In general, this research paper is divided into four sections starting with this introduction. Section 2 outlines the systematic methodological approach including statistical analysis. Section 3 describes the key findings from the research and provides an in-depth discussion to explore the objectives. Section 4 concludes the findings with a discussion of future directions for this topic of study.

## 2 Materials and methods

### 2.1 Experimental site and climate condition

**Fig. 1:**
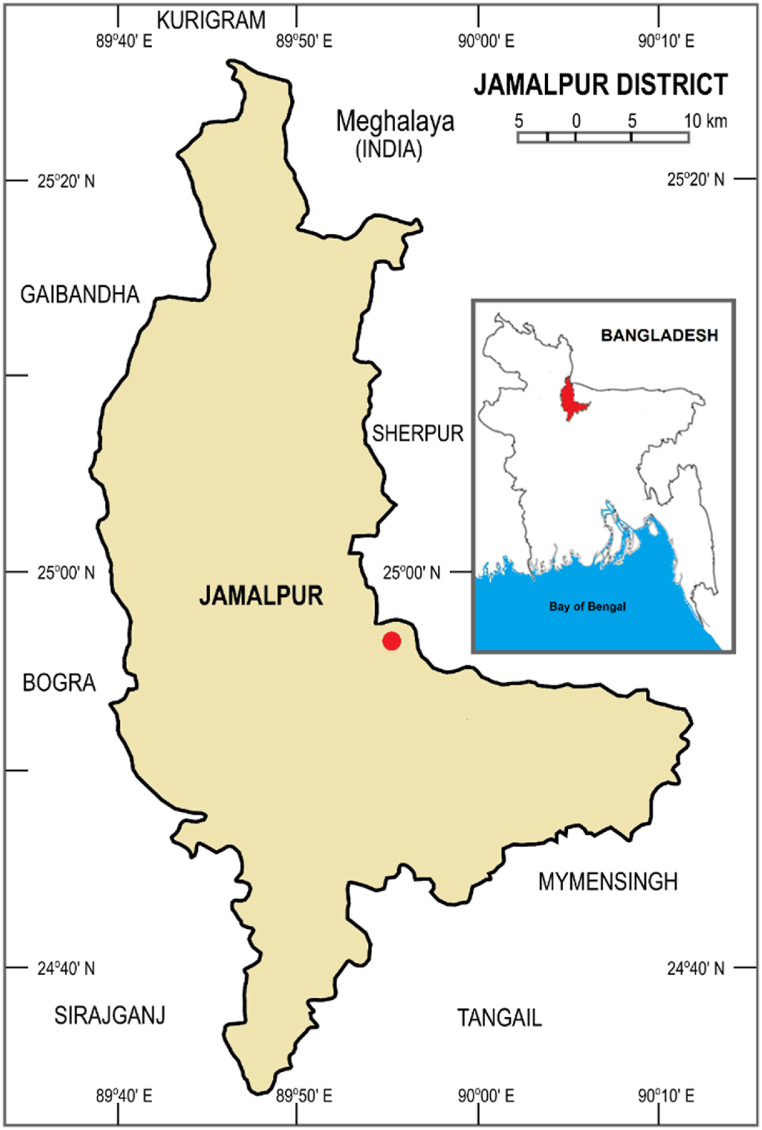
Study area and soil sample location map at Jamalpur, Bangladesh, as indicated by the red dot.

The field experiment was carried out at the Regional Agriculture Research Station (RARS), BARI, in Jamalpur, Bangladesh (Fig. 1). The experiment took place over two consecutive years 2020-21 and 2021-22, during the *Robi* season in the same plots. The experimental soil was located in agroecological zone 28 (Madhupur tract) in Bangladesh. The environmental scenario was characterized by a semi-arid monsoon and sub-tropical climate, with varying levels of rainfall throughout the year. The location of the experimental information and environmental conditions for this study are presented in Table 1.

**Table 1:** Study field characteristics used in onion tillage practices.

Parameters	Details	
Location	Regional Agricultural Research Station (RARS), Jamalpur, Mymensingh division, Bangladesh	
Soil type	Silt clay loam	
Location	24°56′11′′N latitude, 89°55′54′′E longitude	
Altitude	Height above mean sea level: 16.46 m	
Rainfall (avg.)	Monsoon (June–September)	Dry-winter (November–March)
	1549.45 mm	440.00 mm
Drainage	Moderate	
Temperature	32 °C (Max), 20 °C (Min)	

### 2.2 Experimental soil properties and analysis

Soil samples were taken at depths ranging from 0 to 20 cm before tillage, after tillage (shortly before transplanting the onion seedlings), and post-harvest in order to analyze the soil's physiochemical parameters. The physical properties of the soil were analyzed using the standard procedures. The bulk and particle densities were determined using standard procedures [21,22]. The core sampling method was used to determine the soil bulk density (BD). A soil core of a known volume (100 cm³) was collected using a core sampler. The mass was measured ($W_{m}$), then dried in an oven at 105°C for 24 h, and measured again ($W_{d}$). The calculation of BD involved dividing the dry mass ($W_{d}$) by the volume (V) and expressing the result in g cm^−^³ [23].

(1) $$\text{Bulk}\;\text{density},\text{BD}=W_{d}/V\times 10$$

The measurement of soil particle density (PD) was conducted using the pycnometer method. An established quantity of soil (50 g) was carefully placed in a pycnometer, and the volume was measured by displacing water. The calculation for PD involves dividing the mass of soil by the volume of soil particles, and the result is expressed in g cm^−^³. The porosity of the soil then calculated using Equation (2).

(2) Porosity(%)=(1−BD/PD)×100

Determining field capacity (FC) involved saturating a soil sample and allowing it to drain naturally and freely under gravity until no further water was observed to drain. The soil moisture content (MC) at this point was determined using the gravimetric method [24]. The FC is determined by dividing the mass of water retained in the soil by the dry mass of the soil, and is expressed as a percentage. The infiltration rate was determined using a double-ring infiltrometer [25]. Two concentric rings were placed into the soil, and water was added to both rings. The decrease in water level in the inner ring during a specific time period was recorded. The infiltration rate was determined by measuring the water infiltrated the soil over a unit area and unit time period. This measurement is typically expressed in cm hr^−1^.

The MC of the soil was determined using the gravimetric method outlined by Refs. [22,26]. Soil samples were typically collected at a depth of 0–20 cm. The gravimetric soil MC refers to the amount of water present in the soil, determined by measuring the weight difference between the wet soil and the dried soil at a temperature of 105°C for 24 h, also known as the oven-dry weight. Soil sampling was followed for capturing both top surface and slightly below top surface (subsurface) moisture levels, where the onion root can be grown. Soil MC monitoring was carried out: before tillage, pre-transplanting after tillage, immediately post-irrigation, and pre-harvest to understand moisture status as the crop matures. This sampling strategy helps evaluate how different tillage practices affect water retention across the soil profile throughout the crop's growth cycle. The quantity of irrigation water applied was measured by a core sampling method by the following equation:

(3) $$\text{MC}\;\left(\%\right)=\frac{\left(mass\;of\;moist\;soil\;\left(g\right)-mass\;of\;oven\;dried\;soil\;\left(g\right)\right)}{mass\;of\;oven\;dried\;soil\;\left(g\right)}\times 100$$

Data on the amounts of irrigation water applied for each experimental plot were gathered via a core sample, and analysis was used as the standard method using the equation:

(4) $$IR=\frac{FC-MC}{100}\times apparent\;specific\;gravity\;or\;BD\times Root\;zone\;Depth\;\left(cm\right)$$

Where IR is the irrigation water amount (m^3^). Irrigation water use efficiency (WUE) or water productivity index (WPI) is calculated as the ratio of the crop yield generated to the amount of irrigation water consumed, which the equation can define:

(5) WPI=Y/IR

Where Y is the harvested bulb yield (kg).

### 2.3 Experimental design, tillage, irrigation and fertilizer management practices

The experiment was designed using the randomized complete block design (RCBD) method with four replications. The experimental treatments were implemented using various tillage practices and irrigation methods, as shown in Table 2. The experiment consisted of three treatments: namely, i) T_1_ = Minimum tillage (MT) practice using a BARI inclined plate planter [27]; ii) T_2_ = Tractor tillage (TT) practice, where a single tillage operation was performed at once using a tractor (Sonalika Supreme tractor with Sonalika smart rotavator (48 Blades); and iii) T_3_ = Conventional power tillage (PT) practice (farmers practice) using a Power Tiller with 24 tilling blades.

**Table 2:** Different tillage practices in the study.

Crop	Conservation tillage practices	Irrigation method	Residue management
BARI Onion 4 (*Allium cepa* L. cv BARI Piaz-4)	T_1_ = Minimum tillage (MT) practice, single tillage and the simultaneous sowing operation using BARI inclined plate planter [27]	Furrow (Flood) irrigation	Rice crop residue (15 cm)
	T_2_ = Tractor tillage (TT) practice, single tillage performed at once by tractor [28]		
	T_3_ = Conventional power tillage (PT) practice (farmer's practice), typically consists of three to four passes by power tiller followed by two to three ladder passes [29]		

The tillage practices were carried out using particular equipment for each practice, and the rice crop residue from the previous rice crop was pulverized and incorporated into the soil. The tillage practices used for the T_1_ treatment by the BARI inclined plate planter – a special type of two-wheel tractor (2WT), which is also known as a power tiller with 48 tilling blades [27]. The tillage practices used for the T_2_ treatment by a Sonalika Supreme tractor (DI 48 RX model) with Sonalika smart rotavator (48 Blades) [28], and for T_3_ treatment by an ACI Power Tiller (R24 20 HP) with 24 tilling blades [29], which involved three passes of tillage using a power tiller followed by 2–3 laddering.

The BARI Onion-4 (*Allium cepa* L. cv BARI Piaz-4) was employed to transplant the onion seedlings, considering seedlings at 40 days. The plot size was 9 m by 2 m, with a buffer distance of 1 m. The planting spacing in the experiment was kept constant at 15x10 cm. Fertilizer was applied using urea, TSP, MP, and gypsum, with the respective amounts of 240-260-225-110 kg ha^−1^ for N-P-K-S. Additionally, cow dung was applied at a rate of 5 tons ha^−1^, following the fertilizer suggestion guided by the BARC Fertilizer Recommendation Guide-2018 [30]. Half of the nitrogen and potassium (N & K) and the entire amount of other fertilizers were applied as a basal application during the final land preparation. The remaining nutrients were applied as a top dressing during the flowering stage, following irrigation at 50–55 days after transplanting (DAT).

The onion cultivation was adapted as a flatbed method. Irrigation operations were performed four times during each growth stage using a flood irrigation system. Irrigation was applied based on the four growth stages in onion cultivation, namely, (i) after the time of seedling transplanting, (ii) at the time of inflorescence emerges stage, (iii) At the time of flowering stage and (iv) At the time of seed formation stage, with a total of four irrigations applied at each stage. The first irrigation was applied just after transplanting for the plant establishment on the soil, and watering stopped 25 days before harvesting when bulbs reached full size and the tops began to fall [31]. Based on the treatments, each plot received a specific amount of water at regular times to ensure that the soil moisture content in the root zone remained at field capacity. The crop was maintained free of weeds through periodic manual hoeing when necessary.

### 2.4 Data collection and analysis

The data collection process complied with the established protocols outlined for agronomic field experiments, as specified by Ref. [32]. Measurements pertaining to bulb yield characteristics were gathered from a sample of 20 plants selected at random, once the crops completed their maturity stage. This was done by using quadrates at each sub-plot. In order to collect plant and other characteristics data, the onion crop was harvested from the cultivating field and stored in a dry room at room temperature. To obtain dry weight, the collected onion bulbs were sun-dried until a 12% moisture content was achieved. A similar approach was employed for measuring the dry biomass of onions. The yield and yield parameters were determined using standard methods for onion bulb diameter (cm), bulb height (cm) and straw weight (g), and yield (t ha^−1^). Final onion bulb yield and biomass yield data were recorded after the crop harvest on a whole plot basis.

### 2.5 Statistical analysis

The onion yield and yield characteristics obtained from the three treatments and replications were used to calculate the mean standard deviation (SD), standard error (SE), and least significant differences (LSD) (±, if applicable). Statistical analysis of different tillage treatments was performed using the 'R' computer package developed by the R core team [33]. Analysis of variance (ANOVA) tests were used to identify statistically significant differences (at a significance level of α = 0.05) between treatments. Comparisons were made between the means using the Least Significant Difference (LSD) test with a significance level of 5%.

## 3 Results and discussion

### 3.1 Conservation tillage impact on Initial soil physiochemical properties

Table 3 displays the physiochemical characteristics of the experiment field's soil before and after tillage treatments, shortly before transplanting the onion seedlings. Results show that the bulk density (BD) is close to the normal range of 1.50 to 1.47 g cm^−3^ [34] and it increased as the tillage depth increased. The soil bulk density, which is strongly influenced by soil structural characteristics, is dependent on the method of tillage practices and varied tillage practices may modify these properties [35,36]. MT maintains lower bulk density near the surface and avoids compaction at greater depths, thereby promoting better soil structure and aeration.

**Table 3:** Soil's physiochemical properties at the experimental site prior to tillage operation and after tillage operation shortly before transplanting the onion seedlings.

Soil physical properties											
Treatments	Soil depth (cm)	Initial Moisture (%)	After tillage Moisture (%)	Bulk density (g cm^−3^)	Particle density (g cm^−3^)	Porosity (%)	Infiltration (mm h^−1^)	Field capacity (%)	Soil textural class		
									Sand	Silt	clay
Initial soil	0–20	19.6	–	1.40 a	2.62	46.81 d	8.50 b	29.41 c	43.61	34.28	22.11
T_1_	0–20	–	18.5 a	1.37 b	2.61	47.65 a	8.58 a	29.98 a	–	–	–
T_2_	0–20	–	17.6 c	1.39 a	2.61	47.55 b	8.39 c	29. 77 b	–	–	–
T_3_	0–20	–	17.9 b	1.39 a	2.61	47.46 c	8.43 c	29.78 b	–	–	–

CV (%)	–	–	1.24	0.82	–	0.79	0.19	0.24	–	–	–
LS	–	–	LS	LS	NS	LS	LS	LS	–	–	–

Soil chemical properties															
Treatments	Soil depth (cm)	pH	OM (%)	OC (%)	Ca	Mg	K	Total N%	P	S	B	Cu	Fe	Mn	Zn
					m_eq_ 100g^−1^				μg g^−1^						
Initial soil	0–20	6.6	2.1	1.22	6	1.9	0.07	0.1	5.21	8.75	0.32	2.4	20	4	1.22
T1	0–20	6.68	2.31	1.34	6	1.9	0.7	0.12	5.32	9.1	0.31	2.4	18.5	4	1.22
T2	0–20	6.64	1.93	1.16	6	1.9	0.4	0.09	5.23	8.77	0.28	2.3	20.1	4	1.23
T3	0–20	6.64	2.02	1.19	6	1.9	0.6	0.08	4.56	8.72	0.28	2.3	20.2	4	1.22

Critical level	–	6–8	–	–	2	0.5	0.12	–	10	10	0.2	0.2	4	1	0.6
Interpretation	–	O	L	L	O	H	L	VL	L	O	L	VH	H	O	H

Note: T_1_ = Minimum tillage (MT) practice; T_2_ = Tractor tillage (TT) practice; T_3_ = Power tiller (PT) practice (farmers tillage practice). CV = Coefficient of variance, LS = Level of significance, ∗∗ = Significant at p = 0.05, NS = non-significant. L = low, O = optimum, VL = very low, H = high, and VH = very high values in terms of crop growth.

Results reveal that field capacity (FC) and porosity are near the standard value, as the recommended standard field capacity lies between 25 and 35% and porosity 47–51% [37]. It is noted that the FC and porosity are lower for TT and PT practice compared to MT practice [38], while BD is higher. This reduction occurs because intensive tillage practices like those involving tractors and power tillers disrupt soil structure, reduce the number of soil pores, and compact the soil, thereby decreasing its ability to retain water. In contrast, MT preserves soil structure, maintains pore spaces, and enhances water retention, so it requires less irrigation to avoid yield loss hence potentially avoiding a rise in the production expenses associated with CT practices [39].

Table 3 also shows that conservation MT practice increases soil infiltration rate and thereby reduces soil evaporation resulting in increased soil water storage [38,40], contingent upon the particular conditions in the field and the quantity of organic matter there. The soil profile of silt clay loam soil was found at the experimental site, specifically at a 0–20 cm depth. Soil samples were dried at room temperature, crushed, and sieved using a 10 mm sieve for texture assessment. After leveling, samples were securely preserved in polythene bags for lab testing. Hydrometer-based particle size distribution analysis was used to assess soil samples [41]. Results reveal that experimental soil texture classifications are close to previous research study [41]. The sand fraction is relatively high, primarily due to its geographical location along the banks of the Brahmaputra River. Charland is a riverine island and floodplain region, often getting sand from riverine sediments after floods. For this, proper management of the soil has the potential to considerably enhance its productivity.

The MT practices often result in better soil moisture retention, as shown in Table 3. This is because these practices reduce soil disturbance and increase the amount of organic matter in the soil. The moisture content of the soil can vary between 22% and 32% at field capacity, indicating a higher ability to retain water compared to traditional tillage methods [42]. In the experimental site, soil moisture was below the optimum level, necessitating supplementary irrigation to get water available into the plant. The optimum soil moisture range for promoting onion growth is between 50% and field capacity, ensuring that the soil moisture level does not drop below 50% of the total available soil moisture [43].

In soil chemical analysis, soil pH can vary under different tillage practices. Soil pH under MT practice tends to remain more stable, as organic matter is preserved and decomposition occurs slowly. In contrast, tractor and power tiller tillage can lead to lower soil pH due to increased aeration and microbial activity, which accelerates the decomposition of organic matter and nutrient leaching. Overall, intensive tillage practices may cause greater fluctuations in soil pH, potentially affecting nutrient availability and crop growth. Slightly reduced soil organic matter (SOM) was measured in this study. MT practice promotes the accumulation of SOM by minimizing soil disturbance, supporting SI and CSA through enhanced soil fertility and structure [44]. TT and PT tillage, however, can lead to faster decomposition and loss of SOM due to increased soil aeration and erosion. This reduction in SOM under intensive tillage practices may compromise long-term soil health and productivity, making minimum tillage more favorable for sustainable farming.

### 3.2 Conservation tillage impact on post-harvest soil physical properties

Table 4 shows the findings of the post-harvest soil analysis, focusing on the changes in the physical properties of the soil. The findings reveal that the soil moisture content (MC) is often below the field capacity when measured post-harvest. The Table further reveals that the soil is not completely saturated at the start of the growing season, implying that soil moisture after tillage might not be as effective in preserving residual soil moisture at the top soil surface. For the post-irrigation stage, the soil moisture levels exceed the field capacity, indicating a temporary saturation caused by irrigation. The MC after irrigation is highest with minimum tillage (MT), indicating that it could potentially slow down water infiltration and enhance moisture retention in the upper layers. One possible reason for this could be a decrease in soil disturbance, resulting in lower rates of water percolation. The MC for tractor tillage (TT) is lower than that of MT but higher than that of PT practice. This suggests that TT practice has moderate infiltration and retention characteristics, which may maintain a balance between moisture retention and drainage. PT practice shows more quick infiltration and drainage, potentially improving water movement through the soil profile and reducing the time the soil stays saturated.

**Table 4:** The status of the post-harvest soil's physical properties at the experimental site.

Treatments	Soil depth (cm)	Soil moisture (%)			Bulk density (g cm^−3^)	Particle density (g cm^−3^)	Porosity (%)	Infiltration (mm h^−1^)	Field capacity (%)	Textural Class		
		After tillage	Post-irrigation	Pre-harvest						Sand	Silt	Clay
Initial soil	0–20	19.6 a	–	–	1.40 b	2.62	46.81 b	8.50 c	29.41 b	43.61d	34.28 a	22.11 c
T_1_	0–20	18.5 b	32.5 a	23.9 a	1.37 c	2.62	47.47 a	9.25 a	31.80 a	44.30 c	33.94 b	21.76 a
T_2_	0–20	17.6 c	30.6 b	22.5 c	1.45 a	2.62	45.86 c	8.80 b	28.50 c	45.29 b	33.53 c	21.18 b
T_3_	0–20	18.2 b	31.4 c	23.2 b	1.48 b	2.62	45.75 d	8.23 d	27.90 d	45.8 a	33.1 d	21.1 c

CV (%)	–	1.37	1.02	1.13	0.82	–	0.81	0.59	1.44	1.12	0.67	0.26
LS	–	LS	LS	LS	NS	–	NS	NS	NS	LS	LS	LS

Note: T_1_ = Minimum tillage (MT) practice; T_2_ = Tractor tillage (TT) practice; T_3_ = Power tiller (PT) practice (farmers tillage practice). CV = Coefficient of variance, LS = Level of significance, ∗∗ = Significant at p = 0.05, NS = non-significant.

Table 4 illustrates a reduction in bulk density by 1.37 g cm^−3^ and an increase in porosity by 47.47% for MT practice, suggesting enhanced soil structure and aeration capacity. Improved hydrological infiltration (9.25 mm h^−1^) and field capacity (31.80%) indicate enhanced water retention and movement, which is likely beneficial for root development and moisture availability. This technique (MT) demonstrates a favorable alignment with preserving soil health and sustainability. For TT practice, the table demonstrates a marginal rise in bulk density (1.45 g cm^−3^) accompanied by a decrease in porosity (45.86%), suggesting a modest level of soil compaction. The lower infiltration rate of 8.80 mm h^−1^ and field capacity of 28.50% indicate decreased water permeability and retention. This could hinder water availability, therefore affecting overall crop growth in the long run. PT practice treatments had the highest bulk density (1.48 g cm^−3^) and lowest porosity (45.75%), indicating substantial compaction and degradation in soil structure. The observed decrease in infiltration (8.23 mm h^−1^) and field capacity (27.90%) indicates restricted water availability and possible difficulties in crop productivity caused by reduced root penetration and aeration. Overall, MT practice maintains better soil physical characteristics, such as improved water use efficiency and soil health. Conversely, tractor and power tiller tillage methods have adverse effects that can be attributed to compaction. Results show variation in soil texture under MT, TT and PT practices, which is mostly attributed to the different levels of soil disturbance and compaction induced by each practice. MT conserves the original soil structure and aggregates, hence sustaining a stable texture with a balanced porosity. In contrast, TT and PT practices for soil cultivation disturb the structure of the soil, compact it, and alter the particle distribution and pore space, which may impact the physical characteristics of the soil, such as its texture perception and classification over time. For this reason, proper management can significantly enhance the soil texture. Overall, MT practices increase soil porosity, water infiltration, and moisture retention, which are crucial for sustainable intensification by supporting soil health with continuous crop production with minimal environmental impact. Conservation tillage also aligns with climate-smart agriculture by improving SOM, reducing environmental emissions and increasing resilience to climate variability, thus contributing to both productive and sustainable farming systems [39,45].

### 3.3 Different tillage practices and irrigation impact on the bulb yield and yield contributing characteristics

Table 5 displays the onion bulb yield and yield contribution characteristics under different tillage practices and irrigation water usage. The table shows that the tillage practices have an impact on the bulb diameter and productivity of onion bulbs. The average 20 bulb weight differed significantly among the different tillage practices, where the MT practice produced the highest average fresh 20 bulb weight (125 g). In contrast, the PT practice produced the lowest average 20 bulb weight (94 g). The conservation tillage practice of MT practice has shown the highest bulb yield (22.79 t ha^−1^, avg.) and the PT practice showed the lowest yield (16.25 t ha^−1^, avg.). One of the treatment plots of MT practice produced the highest bulb yield of 23.5 t ha^−1^. The results were mostly attributed to the larger diameter and length of the bulb. These findings suggest that the bulb yield and yield contributing characteristics observed in MT practice are affected by the size of the soil clods. Consequently, the fineness and looseness of the soil condition have led to a higher bulb yield when the water amount used remains the same for two consecutive years. On the other hand, a comparatively lower yield was found in PT practice because the soil was pulverized excessively, resulting in loosening the soil nutrients and impacting moisture levels [46], while this practice has reduced onion bulb volume and yield productivity due to increasing moisture stress.

**Table 5:** Yield and yield contribution characteristics of BARI onion-4 under different tillage.

Treatment	Plant population /m^2^	Days of flowering	Days of maturity	Average 20 Bulb diameter (cm)	Average 20 Bulb height (cm)	Fresh 20 Bulb weight (g)	Sundry 20 Bulb weight (g)	Fresh bulb yield (t ha^−1^)	Sundry bulb yield (t/ha)	Fresh Straw Yield (t/ha)	Sundry Straw Yield (t/ha)	Harvest Index
T_1_	88	60	125	6.72 a	5.54 a	125 a	107.59 a	22.79a	20.48a	4.94a	4.15a	0.83
T_2_	83	60	125	6.05 b	5.00 b	95 b	80.56 b	20.48b	17.93b	3.69b	3.10b	0.85
T_3_	84	60	125	6.03 b	4.75 c	94 b	77.97 b	16.25c	14.66c	2.80c	2.35c	0.86

Mean	85	–	–	6.26	5.09	104.63	88.71	19.84	17.69	3.81	3.20	–
CV (%)	1.04	–	–	1.16	2.13	0.94	0.94	2.00	2.89	2.14	1.18	–
LSD (0.05)	0.09	–	–	0.13	0.19	0.08	0.07	0.69	0.88	0.19	0.14	–
LS	ns	–	–	∗∗	∗∗	∗∗	∗∗	∗∗	∗∗	∗∗	∗∗	–

Note: T_1_ = Minimum tillage (MT) practice; T_2_ = Tractor tillage (TT) practice; T_3_ = Power tiller (PT) practice (farmers tillage practice). CV = Coefficient of variance, LS = Level of significance, ∗∗ = Significant at p = 0.05, NS = non-significant.

Harvest index (HI) is used in agronomy to measure the amount of grain yield in relation to the total aboveground biomass of a crop, and its range varies depending on the crop [47]. HI is determined by dividing the economic yield by the entire biomass, which includes bulbs, leaves, and stems, and then expressing the result as a percentage. The results in the Table show a significant rise in HI, with average values of 83%, 85%, and 86% for the MT, TT, and PT practices, respectively. The presence of high HI is associated with the production of high bulb weight rather than the above-ground biomass. This could be attributed to an increased assimilated photosynthate, which could significantly improve bulb productivity and the partitioning of the same to the bulbs [48].

Table results indicate that changing the tillage frequency (i.e., minimum/zero tillage with less soil disturbance) and optimum plant-line spacing could be beneficial in adverse biophysical conditions and may be adopted as a climate change adaptation strategy. CTS practices promote efficient use of limited water resources by minimizing soil disturbance and maintaining surface residues that preserve organic matter and soil moisture, which helps retain moisture and improve root growth and nutrient uptake under water-limited conditions [49], while sustainable intensification focuses on optimizing resource use and maintaining high yields. Together, these practices enhance soil health and resilience to moisture stress, allowing onions to grow more effectively in challenging conditions by improving water use efficiency and reducing the impact of drought or irregular irrigation [50] in locations where there are high chances of drought, or moisture stress may yield better under CTS than conventional TT and PT practice. Nevertheless, the agronomic interventions alone are insufficient to facilitate the adaptation and mitigation of agricultural systems to climate change in Bangladesh. Instead, comprehensive and integrated development programs, including extension and credit, are necessary to support adopting sustainable crop management techniques [51], such as CTS. Enhancing soil health and optimizing water use help achieve higher yields sustainably under challenging conditions. From the above results and discussion, it is evident that the MT practice in the Charland agroecosystem has a significant positive effect on yield productivity in a vulnerable climate ecosystem.

### 3.4 Different tillage practices and irrigation impact on water footprint and optimization of water saving potential

The water footprint (WF) refers to the overall quantity of water utilized in producing a specific food crop on a 1.0 ha of land, commonly expressed as cubic meters per hectare [2]. Table 6 displays the results of water use efficiency or water productivity index (WPI) and WF for various tillage practices. To compute the WF and WPI, evaluating the water used during the entire crop growth cycle is necessary. The WF calculated from this experiment was 5.239, 5.830 and 7.348 m^3^ t^−1^ for the MT, TT and PT practices, respectively. The WF and WPI were noticeably influenced by the treatments (p < 0.05). According to the LSD test (p < 0.05), the WPI of the MT practice (190.86 kg m^−3^) is significantly different from the other tillage treatments since it is statistically lower than the other treatments. The cultivation of onion crops using the MT process resulted in bulb yield levels ranging from 16.25 to 22.79 t ha^−1^, which were, on average, 40% more efficient water use than conventional tillage practice with equal bulb yield. The results of this study are consistent with previous research indicating that in addition to decreasing soil evaporation, the technique of MT is also more effective in optimizing water utilization by onion crops [52,53]. The CA tillage practices aim to manage water in water-deficient agroecosystem onion cultivation settings effectively [39,54]. Given the growing threats of climate change, including more frequent and severe droughts and storms, adopting minimum tillage practices will be crucial in promoting more significant water infiltration, minimizing surface water loss through runoff, and increasing the amount of water available for onion plants [55].

**Table 6:** Assessing water footprint and potential for water conservation across various tillage practices.

Treatments	No. of Irrigation	Amount of Irrigation water (m^3^)				Total water amount (m^3^ plot^−1^)	Total water amount (L plot^−1^)	Effective rainfall (m^3^)	Irrigation water amount (m^3^ ha^−1^)	Fresh bulb yield (t ha^−1^)	Water use efficiency (t m^−3^ha^−1^)	Water footprint (m^3^ t^−1^)	Water productivity index (kg m^−3^)	Water saving (%)
		DAS 2	DAS 25	DAS 60	DAS 90									
T_1_	4	0.044	0.061	0.055	0.055	0.215	214.93	0	119.41	22.79	0.1909	5.239	190.86	40
T_2_	4	0.044	0.061	0.055	0.055	0.215	214.93	0	119.41	20.48	0.1715	5.830	171.51	26
T_3_	4	0.044	0.061	0.055	0.055	0.215	214.93	0	119.41	16.25	0.1361	7.348	136.09	0

Note: T_1_ = Minimum tillage (MT) practice; T_2_ = Tractor tillage (TT) practice; T_3_ = Power tiller (PT) practice (farmers tillage practice).

**Fig. 2:**
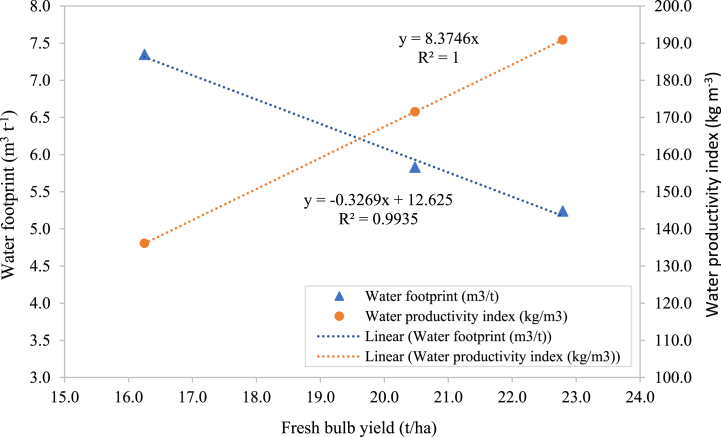
Water footprint and water productivity index (WPI) for onion cultivation in relation to the fresh bulb yield.

Fig. 2 shows the relationship between fresh bulb yield (t/ha), water footprint (WF), and water productivity index (WPI) for onion cultivation. It can be seen that WF of 5.239, 5.83 and 7.348 m^3^ t^−1^ decreased linearly with decreasing levels of onion bulb production yield for minimum, tractor and conventional tillage practices, ranging from 22.79, 20.48 and 16.25 t ha^−1^, respectively, demonstrating that second and third-order polynomial coefficients are not significant (p < 0.05). Results also imply that the minimal 5.239 m^3^ of water used to produce 1 ton of bulb yield is the highest production level among the treatments.

The equation y = −0.3269x+12.625 with R^2^ = 0.9935 indicates a strong negative linear relationship between fresh bulb yield and WF. As the yield increases, the WF decreases significantly, demonstrating that higher yields are associated with more efficient water use. The blue points represent the water footprint, which decreases linearly as the bulb yield increases, indicating more efficient water use with higher yields. The equation y = 8.3746x with R^2^ = 1 shows a perfect positive linear relationship between fresh bulb yield and water productivity index. As bulb yield and WPI increase, it suggests improved efficiency in water use per kilogram of onions produced. The orange points represent the water productivity index, which increases linearly with yield, showing that higher yields correspond to greater productivity per unit of water used. The regression equations and high R^2^ values indicate strong correlations between bulb yield, water footprint, and WPI. From the regression analysis in the figure, the trend suggests that for every increase in yield, there is a significant improvement in WPI or water use efficiency, and optimizing yield positively impacts the WPI.

**Fig. 3:**
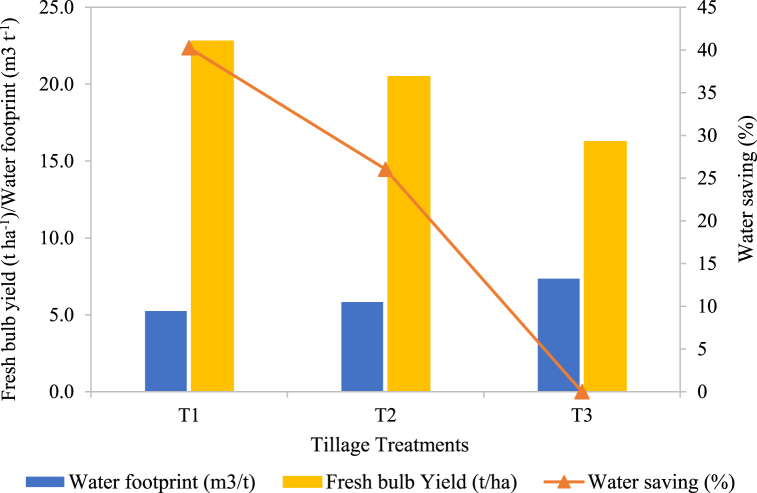
Water footprint and water-saving percentage in relation to the fresh bulb yield. Note: T_1_ = Minimum tillage (PTOS); ii) T_2_ = Tractor tillage practice; iii) T_3_ = Conventional tillage practice (farmers practice).

Fig. 3 shows the relationship between WF, water-saving percentage and fresh bulb yield under different tillage treatments. In the blue bar, WF decreased progressively from MT to PT practices, indicating that PT practice has the lowest water usage per ton of onions produced, reflecting more efficient water use in MT practice. In the yellow bar, bulb yield is highest in MT and slightly decreases in TT and PT practices. Water saving percentage decreases significantly from MT to PT practices in the orange bar. The result suggests that MT practice yields the freshest bulbs and achieves significant water savings potential (over 40%), highlighting that MT practice achieves the highest water-saving which makes it the most efficient in conserving water. This indicates that conservation tillage practices (MT) can substantially reduce water use without significantly compromising bulb productivity, making it a viable strategy for sustainable onion production in water-limited environments.

## 4 Conclusion

The different tillage practices significantly impacted the yield parameters and yield contributing characteristics in onion cultivation within the Charland agroecosystem. Among the different tillage practices, conservation MT practices showed the highest bulb yield, and conventional PT practice showed lower bulb yield productivity. However, each tillage practice demonstrated a statistically different bulb yield productivity. Therefore, MT practice was found to be the most suitable tillage practice for achieving the highest bulb yield of onion.

Moreover, the study evaluated the impact of CT and irrigation on improving soil properties, water footprint (WF) and water productivity index (WPI), taking into account the contributing characteristics of bulb yield and yield contributing matrices. The bulb production and water conservation in MT practice differed from those in other tillage treatments. Nevertheless, there is an inverse relationship between the onion bulb production level and the irrigation water demand. Consequently, the findings suggest that when the surface soil is covered with small amounts of crop residues, it leads to a significant decrease in the water used by onion crops while maintaining high bulb yield. Additionally, there is a notable increase in the WPI when irrigation is applied at the appropriate time and in the correct quantity. This experiment can be fruitfully applied in the vulnerable climate of the Charland agroecosystem and synchronically applied to both MT and irrigation practices, resulting in water-saving potentials that could save soil health with high onion productivity.

Based on the analysis, conservation agriculture, specifically through practices like conservation tillage and sustainable intensification, proves effective in improving WPI or water use efficiency and maintaining crop yields under moisture-stress conditions. The result shows that treatments like MT practice achieve substantial water savings (40%), demonstrating the potential of these methods to optimize resource use. By reducing the water footprint and enhancing water productivity, conservation agriculture supports sustainable agricultural practices, helping ensure high productivity with minimal environmental impact and improved resilience to water scarcity. Future research could focus on the optimization of water-use efficiency and the reduction of the water footprint in onion cultivation, by improving soil health through increased organic carbon sequestration. Furthermore, research should focus on measuring the effects of CT on greenhouse gas emissions and tailoring practices to different regions, especially in smallholder farming systems that can be adapted to local environmental and economic conditions.

## CRediT authorship contribution statement

**Md Mashiur Rahman:** Writing – review & editing, Writing – original draft, Visualization, Validation, Supervision, Software, Resources, Methodology, Investigation, Formal analysis, Data curation, Conceptualization. **Naznin Sultana:** Writing – review & editing, Software, Resources, Methodology, Formal analysis, Data curation. **Muhammad Arshadul Hoque:** Writing – review & editing, Validation, Supervision, Project administration, Methodology, Funding acquisition, Conceptualization. **Md. Golam Azam:** Writing – review & editing, Visualization, Validation, Methodology, Conceptualization. **Md. Rafiqul Islam:** Writing – review & editing, Visualization, Validation, Software, Methodology, Conceptualization. **Md. Altaf Hossain:** Writing – review & editing, Supervision, Project administration, Methodology, Funding acquisition, Conceptualization.

## Data availability statement

The datasets used and/or analyzed in this study can possibly be obtained from the corresponding author upon a reasonable request.

## Funding statement

This research study was funded by the core research program of the 10.13039/501100005867Bangladesh Agricultural Research Institute.

## Declaration of competing interest

The authors declare that they have no known competing financial interests or personal relationships that could have appeared to influence the work reported in this paper.
